# Methylene Blue Reduces Fluid Loading and Norepinephrine Requirements for Post-Resuscitation Syndrome in a Pig Model of Refractory Cardiac Arrest Resuscitated with Veno-Arterial ECMO

**DOI:** 10.3390/jcm11092515

**Published:** 2022-04-29

**Authors:** Benjamin Pequignot, Mickael Lescroart, Sophie Orlowski, Nathan Reynette, Bana Martini, Eliane Albuisson, Héloise Pina, N’Guyen Tran, Daniel Grandmougin, Bruno Levy

**Affiliations:** 1 Service de Medecine Intensive et Réanimation, Hôpital Brabois, CHRU Nancy, 54500 Vandoeuvre les Nancy, France; bpequignot@hotmail.fr (B.P.); lescroartmickael@gmail.com (M.L.); 2 INSERM U 1116, Equipe 2, Groupe Choc, Faculté de Médecine, 54500 Vandoeuvre les Nancy, France; s.orlowski@chru-nancy.fr (S.O.); d.grandmougin@me.com (D.G.); 3 Faculté de Médecine, Université de Lorraine, 54000 Nancy, France; e.albuisson@chru-nancy.fr (E.A.); nguyen.tran@univ-lorraine.fr (N.T.); 4 Service de Biochimie, Pôle Laboratoires Hôpital Central, CHRU de Nancy, 54000 Nancy, France; 5 Ecole de Chirurgie, Faculté de Médecine, Université de Lorraine, 54000 Nancy, France; nathan.reynette@gmail.com (N.R.); bana.m.98@hotmail.com (B.M.); 6 Plateforme d’Aide à la Recherche Clinique (PARC), ESPRI-Biobase, Hôpital de Brabois, CHRU de Nancy, 54500 Vandoeuvre les Nancy, France; 7 Département d’Anatomie Pathologique, Laboratoires de Biologie Médicale et de Biopatholgie, Hôpital Brabois, CHRU de Nancy, 54500 Vandoeuvre les Nancy, France; h.pina@hotmail.fr; 8 Service de Chirurgie Cardiaque, Hôpital Brabois, CHRU Nancy, 54500 Vandoeuvre les Nancy, France

**Keywords:** cardiopulmonary resuscitation, advanced cardiac life support, out-of-hospital cardiac arrest, extracorporeal membrane oxygenation, vasopressor, microcirculation, inflammatory response, methylene blue

## Abstract

Background: Refractory cardiac arrest management relies on extracorporeal cardiopulmonary resuscitation (ECPR), requiring the use of veno-arterial extracorporeal membrane oxygenation (VA-ECMO). Circulatory flow recovery can be associated with an ischemia–reperfusion injury, leading to vasoplegia and vasopressor requirement. The aim of this work was to evaluate the impact on hemodynamics of a methylene blue bolus infusion in a porcine model of ischemic refractory cardiac arrest. Methods: Ischemic refractory cardiac arrest was induced in 20 pigs. After a low flow period of 30 min, VA-ECMO was initiated and the pigs were randomly assigned to the standard care group (norepinephrine + crystalloids) or methylene blue group (IV 2 mg·kg^−1^ bolus of methylene blue over 30 min + norepinephrine and crystalloids). Macrocirculatory parameters and lactate clearance were measured. Sublingual microcirculation was evaluated with sidestream dark field (SDF) imaging. The severity of the ischemic digestive lesions was assessed according to the histologic Chiu/Park scale. Results: Eighteen pigs were included. The total crystalloid load (5000 (6000–8000) mL vs. 17,000 (10,000–19,000) mL, *p* = 0.007, methylene blue vs. standard care group) and catecholamine requirements (0.31 (0.14–0.44) μg·kg^−1^·min^−1^ vs. 2.32 (1.17–5.55) μg·kg^−1^·min^−1^, methylene blue vs. standard care group, *p* = 0.004) were significantly reduced in the methylene blue group. There were no significant between-group differences in lactate clearance, sublingual capillary microvascular parameters assessed by SDF or histologic Chiu/Park scale. Conclusions: In our refractory cardiac arrest porcine model treated with ECPR, methylene blue markedly reduced fluid loading and norepinephrine requirements in comparison to standard care during the first 6 h of VA-ECMO.

## 1. Introduction

Prolonged refractory cardiac arrest (i.e., without return of spontaneous circulation in the field), is associated with poor outcomes [1]. Temporary replacement of the failing circulation by veno-arterial extracorporeal membrane oxygenation under cardiac massage (VA-ECMO), a strategy called extracorporeal cardiopulmonary resuscitation (ECPR), has been recognized as a potential approach to improve prognosis in selected patients with refractory cardiac arrest [2,3,4]. After prolonged low flow, circulation recovery leads to a post-resuscitation syndrome [5]. First reported by Negovsky in 1972, post-resuscitation syndrome is a complex issue resulting from an ischemia/reperfusion syndrome, and myocardial failure leading to a mixed shock [6]. Circulatory flow recovery increases blood radical oxygen species (ROS), induces excessive nitric oxide (NO) production and can lead to endothelial damage and multiple organ failure (MOF). Critical care management of post-resuscitation syndrome in the first hours following VA-ECMO implantation is mainly based on massive fluid administration, high doses of vasopressor agents (norepinephrine/vasopressin) to provide an adequate mean arterial pressure (MAP), and maximal pump blood flow until hemodynamic stabilization and organ injury recovery [7]. Norepinephrine is the commonly used vasopressor, although high dosages may be associated with several side effects, including on lactate metabolism, tissue perfusion, vascular permeability, and inflammation [8]. Hence, using multiple vasopressors may be necessary to obtain and maintain the hemodynamic target, such as catecholamine-sparing agents, the most commonly used being vasopressin and angiotensin II [9]. The overproduction of NO by soluble guanylate cyclase (sGC) in vascular smooth muscle due to ROS leads to vasodilation and decreased catecholamine activity [10]. Methylene blue (MB) represents an additional adjunct option by its action on NO synthase and sGC activity. In the last decade, MB has been used during septic shock, cardiac surgery vasoplegia and anaphylaxis. Globally, MB use has been associated with increased MAP and systemic vascular resistance (SVR) without any impact on mortality [11,12,13].

The hypothesis of this study is that early MB administration, by blocking sGC, can improve the management of post-resuscitation shock in ECPR resuscitated ischemic cardiogenic shock in pigs. Using a previously published model of post-resuscitation shock [14], this study aimed to assess the impact of MB on the amount of infused fluid, norepinephrine dose, lactate clearance and sublingual microcirculation in the first six hours after VA-ECMO initiation. 

## 2. Materials and Methods

### 2.1. Ethics 

All procedures were conducted in accordance with the European Directive 2010/63/UE related to the Guide for the Care and Use of Laboratory Animals. All experiments were reviewed and approved by the Nancy University Ethics Committee for Animal Experimentation (APAFIS Number 2019112611132983). The procedure for the care and sacrifice of study animals was in accordance with the European Community Standards on the Care and Use of Laboratory Animals.

### 2.2. Animal Preparation

The experimental procedure was performed as previously published, with the same trained surgical team [14,15]. After a one-day fast, animals were placed under general anesthesia and ventilation. Anesthesia was induced via the lateral auricular vein with an intravenous bolus of propofol (1 mg·kg^−1^, Propofol-lipuro 1%, B. Braun, Melsungen AG, Germany). Animals were intubated (TeleflexIsis 7.5 I.D. mm, Teleflex Medical, Athlone, Ireland) and mechanically ventilated (Evita 1 Dura, Dräger, Luebeck, Germany) in assisted-controlled mode (inspiratory fraction of oxygen (FiO_2_), tidal volume 10 mg·kg^−1^ and respiratory rate 12 per minute). Anesthesia was maintained throughout the entire experiment with a continuous infusion of sufentanil (0.2 μg·kg^−1^·min^−1^, sufentanil, Mylan, Canonsburg, PA, USA), propofol (7 mg·kg^−1^·h^−1^, propofol-lipuro 2%, B. Braun Melsungen AG, Germany) and cisatracurium (0.9 mg·kg^−1^·h^−1^, Nimbex, GlaxoSmithKline, Middlesex, Brentford, UK). The animals were monitored by continuous electrocardiogram (ECG) recording. An arterial catheter (5 Fr, Seldicath, Plastimed Prodimed^®^, Neuilly en Thelle, France) was inserted into the right carotid artery for the continuous measurement of systemic blood pressure, while the left carotid artery was dissected and a Transit Time Flow probe (Transonic Systems Inc., Ithaca, NY, USA) secured around the latter. Rectal temperature was monitored and maintained at 38.5 °C. Data were computed using a dedicated analysis program (IOX 2.4.2.6^®^, EMKA Technologies, Paris, France). A triple lumen catheter (8 Fr, Arrow^®^, Reading, PA, USA) was inserted into the right external jugular vein. The right femoral artery was dissected and a femoral arterial outflow cannula (15 Fr, Biomedicus cannulae, Medtronic, Minneapolis, MN, USA) was inserted into the artery. A venous inflow cannula (atriocaval cannula, Edwards, Lifescience, Irvine, CA, USA) was surgically inserted in the right atrium. The pericardium, sternum, and wound layers were closed. A 100 IU·kg^−1^ dose of Heparin (Heparin Sodique Choay, Sanofi-Aventis, Paris, France) was administered prior to the cannulation phase, followed by a continuous IV infusion of 50 IULkg^−1^·h^−1^ to maintain an activated clotting time of 180–250 s. Values were monitored every hour using the Hemochron Jr Signature Microcoagulation System (ITC, Hudsonville, MI, USA). The ECMO consisted of a console (Rotaflow Console, Maquet, Rastatt, Germany), a centrifugal pump (Rotaflow Centrifugal Pump System, Maquet, Rastatt, Germany) and circuit tubing together with the membrane oxygenator (PLS-i oxygenator, Maquet, Rastatt, Germany) linked to a mechanical gas blender system (Sechrist Model 20090, Sechrist, Anaheim, CA, USA). The fully assembled ECMO circuit was primed with saline solution (NaCl 0.9%, B. Braun Medical, Saint-Cloud, France). The oxygen/air flow was repeatedly adjusted to maintain PaCO_2_ and PaO_2_ in the ranges of 4.0–6.5 kPa and 10–15 kPa, respectively, in blood exiting the oxygenator. The left anterior descending (LAD) coronary artery was isolated and a prolene (5/0, Ethicon, Bridgewater, NJ, USA) snare was placed for temporary ligation. After a low median laparotomy, a vesical catheter was placed to estimate hourly urine. Core body temperature was measured via a rectal probe and maintained at 38.5 °C.

### 2.3. Measured Parameters 

#### 2.3.1. Timing of the Measurements

Measurements were performed after surgery at baseline (T_B_), after 30 min of refractory cardiac arrest (at VA-ECMO initiation) (T_0_), after three hours (after randomization) (T_3_) and after six hours (at the end of study) (T_6_).

#### 2.3.2. Hemodynamic Measurements

Heart rate (HR), systolic arterial pressure (SAP), diastolic arterial pressure (DAP) and MAP were continuously recorded.

#### 2.3.3. Microcirculation

Sublingual microvascular circulation was evaluated by a sidestream dark field (SDF) imaging device (Microscan, MicroVision Medical, Amsterdam, The Netherlands) according to published guidelines [16]. The obtained video clips were analyzed semi-automatically using the Automated Vascular Analysis software (AVA 3.0 software, MicroVision Medical, The Netherlands) from which the following microcirculation parameters were collected: total and perfused vessel density (TVD, PVD), proportion of perfused vessels (PPV) and microvascular flow index (MFI). Regional cerebral tissue oxygen saturation (rSO_2_) in the right frontal lobe was monitored by a near-infrared spectrometer (NIRS) device (Inspectra tissue spectrometer Model 650, Hutchinson, MN, USA).

#### 2.3.4. Laboratory Measurements 

Arterial blood gas was assessed in an acid-base and co-oximeter analyzer at central temperature (VetStat, IDEXX Laboratories, Saint-Denis, France). Lactate concentration was measured using a biochemistry analyzer (VetStat, IDEXX Laboratories, Saint-Denis, France). Plasma creatinine, aspartate aminotransferase (ASAT) and alanine transaminase (ALAT), as well as blood urea nitrogen, were measured using an automatic Atellica Solution (Siemens Healthineers, Erlangen, Germany). Lactate clearance was calculated according to the following formula (lactate T_0_-lactate T_6_/lactate T_0_) and expressed as %.

#### 2.3.5. Histology 

Ischemic enteric damage was histologically assessed. Proximal jejunum was sampled at the end of the experiment and fixed in a 10% formalin solution. Fixed tissues were embedded and sectioned for histological analysis. Slides were stained with hematoxylin, eosin and saffron (HES). The Park/Chiu scale, graded from 0 to 8, was used to assess the severity of ischemic digestive lesions.

After animal sacrifice, the bloodless wet weight of the right lung was determined, and its dry weight was also measured after dehydration of the lobe during 24 h in an oven at 200 °C. The wet/dry weight ratio was used as an estimate of pulmonary vascular leakage.

### 2.4. Experimental Protocol 

#### 2.4.1. Cardiac Arrest Model 

After sternotomy, the proximal ligation of the LAD artery induced myocardial infarction leading within minutes toward cardiac arrest in ventricular fibrillation. After a 90-s no-flow period, the animals were resuscitated according to the same resuscitation protocol: an internal cardiac massage was performed and up to 3 internal defibrillations were first attempted at 20 joules. Each defibrillation cycle was followed by two minutes of internal massage at 100 min^−1^. In all instances, ventilation parameters were: tidal volume 10 mL·kg^−1^; respiratory rate 10 per minute; FiO_2_ = 100%; with no positive end-expiratory pressure (PEEP).

After 30 min of CPR, VA-ECMO was initiated at standard-blood-flow 65–70 mL·kg^−1^·min^−1^ based on a theoretical cardiac output and ELSO recommendations. This timing is compatible to ECMO implantation during in-hospital cardiac arrest [17]. Reperfusion of LAD was performed after 30 min of VA-ECMO initiation. At the end of the experiment, animals were euthanized in stopping ECMO.

#### 2.4.2. Hemodynamic Management after VA-ECMO Initiation

MAP was maintained at approximately 65 mmHg using an intravenous form of norepinephrine (Renaudin, Itxassou, France), in which 1 mL of solution contained 2 mg of norepinephrine tartrate, which is equivalent to 1 mg norepinephrine base [18]. Volume expansion (NaCl 0.9%, B. Braun Medical, France) was used in case of decreased blood flow, or in case of jerking or shaking movements of the cannulae.

#### 2.4.3. Groups

Animals were randomly assigned into two groups at the time of ECMO initiation:

(1) Standard care resuscitation group: fluid resuscitation plus norepinephrine. Norepinephrine administration was initiated at a dose of 0.2 μg·kg^−1^·min^−1^ and then increased incrementally by 0.1 μg·kg^−1^·min^−1^.

(2) MB group: fluid resuscitation plus norepinephrine as described above combined with a bolus of 2 mg·kg^−1^ of intravenous MB (ProveBlue^®^, Provepharm, Marseille, France) perfused over the first 30 min after VA-ECMO initiation. 

#### 2.4.4. Exclusion Criteria 

Animals were excluded if a major hemorrhagic event occurred before randomization, if cardiac arrest occurred before baseline measurements, or if the ECMO device failed to provide the theoretical pump flow. 

### 2.5. Data Analysis and Statistics 

Given the small sample size, all results are expressed as median and interquartile range (IQR). Comparisons between two groups were analyzed using the nonparametric Mann–Whitney test. The Friedman test was used for one-way repeated measures analysis of variance by ranks. All statistical analyses were with a significance level of 0.05 and performed using R version 4.0.1 for MacOS^®^ (https://www.r-project.org/, accessed on 15 March 2020).

## 3. Results

Twenty pigs were studied. Two animals were excluded prior to randomization (one for significant hemorrhage, one for refractory ventricular fibrillation prior to baseline measurements). Therefore, 18 pigs were ultimately included: nine in the standard group, nine in the MB group. Morphological, hemodynamic and metabolic parameters, as well as biology and microcirculation SDF, were similar at baseline for the two groups (T_B_) (Tables S1 and S2).

### 3.1. Model Characterization

Characteristics at T_0_ are presented in Table 1 without any difference between the two groups. In both groups time from LAD ligation to cardiac arrest was the same (9.1 (3.5–10.2) minutes in the MB group vs. 7.5 (4.6–11.8) minutes in the standard group, *p* = 0.5). The initial rhythm was ventricular fibrillation for all animals. No differences in no-flow or low-flow were observed between the two groups and ROSC was never achieved in any of the animals studied (Table 1).

### 3.2. Methylene Blue versus Standard Care 

During the 6 h of the study, ECMO-flow and MAP targets were achieved in both groups. Cardiac rhythm remained in asystole or ventricular fibrillation during the entire procedure.

**Figure 1:**
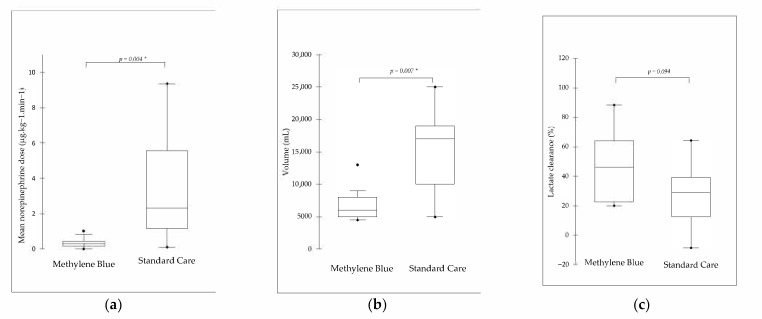
(**a**) Total crystalloid volume perfused (mL); (**b**) mean norepinephrine dose received over the 6 h of veno-arterial extracorporeal membrane oxygenation run (μg·kg^−1^·min^−1^) and (**c**) lactate clearance (%). Data are presented as a median (25^th^–75^th^ percentile). *: Mann–Whitney significance between groups (alpha = 0.05).

The total infused fluid over 6 h was significantly lower in the MB group vs. the standard care group (5000 (6000–8000) mL vs. 17,000 (10,000–19,000) mL, *p* = 0.007). The mean norepinephrine dose over 6 h (μg·kg^−1^·min^−1^) needed to maintain MAP was significantly lower in the MB group than in the standard group (0.31 (0.14–0.44) μg·kg^−1^·min^−1^ vs. 2.32 (1.17–5.55) μg·kg^−1^·min^−1^, *p* = 0.004) (Figure 1).

Median lactate clearance between T_0_ and T_6_ was 29.1 (12.5–39.3) % in the standard group and 46.1 (22.6–64.5) % in the BM group (*p* = 0.09). After VA-ECMO implementation, no between-group differences were recorded for sublingual microcirculatory parameters. The MFI value was significantly lower in the two groups at T_6_ compared to the T_B_ (*p* < 0.001) (Table S2). RSO_2_ in the right frontal lobe was comparable between the two groups throughout the experiment (Figure S1).

**Figure 2:**
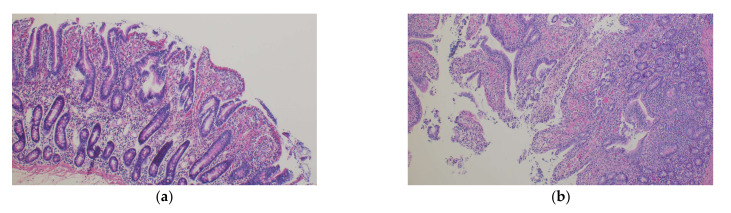
Histology of proximal jejunum samples. (**a**) Methylene blue group, Chiu and Park score of 3, massive epithelial detachment along the sides of the villi, some denuded tips; (**b**) standard care group, Chiu and Park score of 4, denuded villi, dilated capillaries (HES, 100×).

Laboratory measurements at T_6_ are presented in Table 2. Bicarbonate level and pH were significantly higher in the MB group compared to the standard care group at T_6_ (*p* = 0.01). There were no differences regarding ASAT, ALAT plasma creatinine or blood urea nitrogen levels. There was also no significant difference in ischemic enteric damage between groups assessed by the Park/Chiu score (*p* = 0.59) or in lung vascular permeability assessed by lung wet/dry weight ratio (*p* = 0.11) (Table 2 and Figure 2).

## 4. Discussion

The main finding of the present study is that the use of a bolus of MB at the early phase of ECPR markedly reduced the amount of fluid and vasopressor infused in our model of refractory cardiac arrest treated with VA-ECMO. Importantly, the use of MB was not associated with any deleterious effects on microcirculatory parameters, ischemic enteric damage or lactate clearance.

*Shock model*. Similar to bedside observations, all animals at T_0_ presented high lactate level linked to a prolonged cardiac arrest [16]. The abrupt recovery in systemic flow due to the ECMO device was associated with a severe post-resuscitation syndrome requiring vasopressor and fluid administration [17].

*MB improved the hemodynamic*. Our results suggest an additive vasopressor effect in the MB group reflected by the lower mean dose of norepinephrine required to obtain the targeted MAP (2.32 μg·kg^−1^·min^−1^ in the standard group vs. 0.31 μg·kg^−1^·min^−1^ in the MB group). By decreasing the activity of sGC in vascular smooth muscle, MB decreases the vasorelaxant effect of NO. Similar hemodynamic effects (increase in MAP and SVR) associated with a sharp reduction in catecholamine vasopressor requirements were also previously reported during septic shock and post cardiac surgery vasoplegia [19].

*MB use is associated with less fluid expansion*. There are conflicting results regarding the effects of MB on fluid expansion reduction. Donati et al. demonstrated that despite the increase in MAP associated with MB in septic shock patients, these effects were not associated with changes in cardiac diastolic function, pulmonary vascular permeability, or blood volume [12]. Conversely, in a rat model of hemorrhagic shock, MB reduced the volumes of shed blood and of fluids required, and moderated the reduction in packed cell volume, particularly during hypotensive resuscitation [20]. In our study, using the same MAP and VA-ECMO blood flow target, as well as the same markers of hypovolemia, the use of MB was associated with a marked reduction in volume expansion. This can be explained by the action of MB on the sGC pathway which decreases vascular permeability [21,22]. This latter point is crucial since excess fluid balance has been demonstrated to be associated with mortality and morbidity during VA-ECMO [23].

*MB does not improve or worsen microcirculation and lactate clearance*. The effects of MB, which has a powerful vasopressor action on microcirculation, are difficult to anticipate. On the one hand, the reduced perfusion in norepinephrine and amount of fluid infusion may be associated with improved microcirculation, while, on the other, MB also inhibits NO synthesis which is a major regulator of both microcirculatory perfusion and immunomodulation [24,25]. In our study, sublingual microcirculation, including MFI assessed by SDF, was not exacerbated in the MB group when compared to the standard group, nor was the histological ischemic enteric damage. Importantly, hepatic and renal biomarkers were similar in the two groups. Finally, despite the sparing of catecholamine agent and reduction in fluid, lactate clearance remained the same in both groups. However, as stated by Hernandez et al., lactate clearance is not the best marker of perfusion adequation, bioenergetics or tissue protection [26].

*Study limitations*. The first limitation is the apparent small sample size in each group. However, the majority of published studies using a porcine model of refractory cardiac arrest also involved a small number of animals, and this is in keeping with ethical concerns of maintaining the number of animals to the minimum necessary to achieve the aims of the research. Despite coronary reperfusion, no ROSC was achieved for any of the animals in the experiment, which may limit clinical comparability. Finally, the experimental period of six hours is likely too short to allow drawing definitive conclusions for clinical practice, although it does provide precious insights into the early resuscitation phase.

## 5. Conclusions

In the first 6 h of a model of refractory cardiogenic shock treated with ECPR, a bolus of MB markedly decreased catecholamine requirement and the amount of delivered fluid. Conversely, MB did not impact microcirculation parameters, enteric ischemia or lactate clearance.

## Figures and Tables

**Table 1:** Characteristics after cardiac arrest and veno-arterial extracorporeal membrane oxygenation initiation (T_0_).

	Standard Care (*n* = 9)	Methylene Blue (*n* = 9)	*p* Value
No flow (seconds)	91 (88–92)	90 (89–91)	0.41
Low flow (minutes)	31 (29–32)	30 (28–31)	0.51
ROSC	0	0	
MAP (mmHg)	61 (58–65)	68 (65–70)	0.25
pH	7.03 (6.93–7.11)	7.17 7.07–7.21)	0.19
PaCO_2_ (mmHg)	56 (46–63)	48 (43–60)	0.58
PaO_2_ (mmHg)	134 (105–190)	96 (82–180)	0.71
HCO_3_^−^ (mmol/L)	14 (13–15)	15 (14–16)	0.43
Hemoglobin (g/dL)	9.4 (8.8–9.9)	9.7 (8.9–10.6)	0.56
Lactate (mmol/L)	11.6 (10.8–13.2)	10.0 (9.2–10.2)	0.16
ALAT (UI/L)	72 (56–86)	60 (59–75)	0.32
ASAT (UI/L)	35 (35–38)	36 (35–45)	0.68
Creatininemia (mmol/L)	110 (96–115)	106.9 (103–109)	0.35
Urea (mmol/L)	3.7 (3.1–3.9)	3.2 (3–3.3)	0.39

ROCS: Return to spontaneous circulation; MAP: mean arterial pressure; ALAT: alanine aminotransferase; ASAT: aspartate aminotransferase. Data are presented as a median (25th–75th percentile).

**Table 2:** Laboratory and histological data after 6 h, at the end of the experiment (T_6_).

	Standard Care (*n* = 9)	Methylene Blue (*n* = 9)	*p* Value
**Laboratory data**			
pH	7.02 (6.97–7.04)	7.22 (7.1–7.25)	0.01 *
PaCO_2_ (mmHg)	31 (27–42)	40 (37–46)	0.18
PaO_2_ (mmHg)	178 (106–198)	128 (114–200)	0.38
HCO_3_^−^ (mmol/L)	6.6 (6–9)	15 (12.8–17.23)	0.01 *
Hemoglobin (g/dL)	5 (7.4–7.5)	7.9 (7.5–8.8)	0.15
SaO_2_ (%)	94 (92–96)	93 (91–94)	0.55
ALAT (UI/L)	880 (705–991)	1100 (1004–1346)	0.21
ASAT (UI/L)	49 (36–60)	70 (43–81)	0.16
Na (mmol/L)	145 (142–146)	145 (143–146)	0.88
Cl (mmol/L)	117 (112–122)	115 (114–116)	0.30
K (mmol/L)	6.5 (5.9–7.3)	6.2 (5.5–7.2)	0.62
Creatininemia (µmol/L)	130 (107–157)	146 (141–175)	0.19
Urea (mmol/L)	3.9 (3.0–4.8)	4.0 (3.4–4.8)	0.98
Proteins (g/L)	20 (15–22)	24 (23–28)	0.14
Lactate (mmol/L)	8.4 (6.5–10.0)	6.7 (4.5–8.2)	0.10
**Histological data**			
Chiu/Park scale	4 (0–5)	3 (0–3)	0.64
Lung wet/dry weight ratio	4.2 (3.70–6.21)	3.55 (2.58–4.31)	0.11

ALAT: alanine aminotransferase; ASAT: aspartate aminotransferase. Data are presented as a median (25th–75th percentile). *: Mann–Whitney significance between groups (alpha = 0.05).

## Data Availability

Data available on request.

## Supplementary Materials

The following supporting information can be downloaded at: https://www.mdpi.com/article/10.3390/jcm11092515/s1, Figure S1: Regional cerebral regional tissue oxygen saturation (rSO_2_) in the right frontal lobe during study; Table S1: Baseline characteristics of the studied animals (TB). Table S2: Sublingual microcirculation parameters assessed by Microscan.
