# Supplementary material for: Methylene Blue Reduces Fluid Loading and Norepinephrine Requirements for Post-Resuscitation Syndrome in a Pig Model of Refractory Cardiac Arrest Resuscitated with Veno-Arterial ECMO
Source: J Clin Med. 2022 Apr 29;11(9):2515. doi: 10.3390/jcm11092515 (PMC9100142; doi:10.3390/jcm11092515)
Supplement: Supplementary file 1 [file jcm-11-02515-s001.zip › jcm-1686793-supplementary.pdf]

Table S1. Baseline characteristics of studied animals (T_B).

	Standard Care (n = 9)	Methylene Blue (n = 9)	p
Weight (kg)	61 (59 – 65)	60 (55 – 65)	0.37
Heart rate (bpm)	96 (90 – 105)	94 (90 – 107)	0.51
MAP (mmHg)	97 (93 – 111)	115 (110 – 120)	0.45
pH	7.44 (7.42 – 7.49)	7.42 (7.40 – 7.49)	0.31
PaCO2 (mmHg)	42 (37 – 45)	47 (41 – 50)	0.19
PaO2 (mmHg)	179 (161 – 229)	128 (114 – 200)	0.18
HCO3- (mmol/l)	26 (26 – 27)	27 (26 – 28)	0.16
Hemoglobin (g/dL)	10.0 (9.5 – 11.2)	10.2 (9.6 – 10.5)	0.93
Lactate (mmol/l)	1.1 (1.0 – 1.5)	1.2 (1.1 – 1.3)	0.51
ALAT (UI/L)	45 (42 – 51)	50 (49 – 52)	0.34
ASAT (UI/L)	42 (32 – 45)	43 (37 – 67)	0.28
Creatininemia (µmol/l)	87 (77 – 91)	90 (89 – 100)	0.30
Urea (mmol/l)	3.4 (2.7 – 3.8)	3.2 (2.8 – 3.4)	0.59
Proteins (g/L)	53 (51 – 56)	56 (51 – 56)	0.41

MAP: mean arterial pressure. Data are presented as median (25th – 75th percentile). ALAT: alanine aminotransferase; ASAT: aspartate aminotransferase. Data are presented as median (25th – 75th percentile).

Table S2. Sublingual microcirculation parameters assessed by Microscan.

Time	Standard Care (n = 9)	Methylene Blue (n=9)	Standard vs MB
		**TVD (mm/mm²)**	
T_B	20.3 (19.7 – 26.2)	21.9 (19.8 – 23.7)	p = 0.98
T_0	20.6 (20.0 – 21.4)	20.2 (18.8 – 22.3)	p = 0.90
T_3	19.9 (18.5 – 20.2)	17.3 (15.5 – 20.8)	p = 0.35
T_6	20.8 (18.4 – 22.3)	20.1 (18.4 – 21.5)	p = 0.84
**T_B vs T_6**	p = 0.51	p = 0.34	
		**PVD (mm/mm²)**	
T_B	21.1 (20.1 – 26.3)	21.6 (19.7 – 23.2)	p = 0.79
T_0	21.1 (19.8 – 21.1)	21.1 (20.6 – 23.9)	p = 0.30
T_3	19.8 (16.7 – 20.2)	18.7 (15.9 – 21.8)	p = 0.59
T_6	19.2 (18.3 – 20.9)	20.2 (19.3 – 21.7)	p = 0.30
**T_B vs T_6**	p = 0.41	p = 0.63	
		**PPV (%)**	
T_B	98.2 (96.1 – 99.6)	96.7 (95.7 – 98.1)	p = 0.46
T_0	97.2 (94.1 – 99.6)	99.4 (97.3 – 99.1)	p = 0.83
T_3	98.4 (88.1 – 99.4)	97.5 (95.1 – 98.3)	p = 0.97
T_6	97.7 (96.2 – 99.0)	98.8 (96.3 – 99.0)	p = 0.95
**T_B vs T_6**	p = 0.21	p = 0.55	
		**MFI**	
T_B	3.2 (3.0 – 3.5)	3.2 (3.9 – 3.2)	p = 0.64
T_0	2.5 (2.2 – 2.7)	2.5 (2.2 – 2.8)	p = 0.83
T_3	1.9 (1.2 – 2.2)	2.3 (1.8 – 3)	p = 0.14
T_6	2.2 (2.1 – 2.2)	2.4 (1.9 – 2.7)	p = 0.62
**T_B vs T_6**	**$p < 0.05$**	**$p < 0.05$**	

T_B: basal time; T_0: start of VA-ECMO; T_3: 3 hours after randomization; T_6: 6 hours after randomization; TVD: total vessel density; PVD: perfused vessel density; PPV: percentage of perfused vessels; MFI: microvascular flow index. Standard vs. MB groups were compared with Mann-Whitney test and T_B vs. T_6 was compared with Friedmann test. Data are presented as median (25th – 75th percentile).

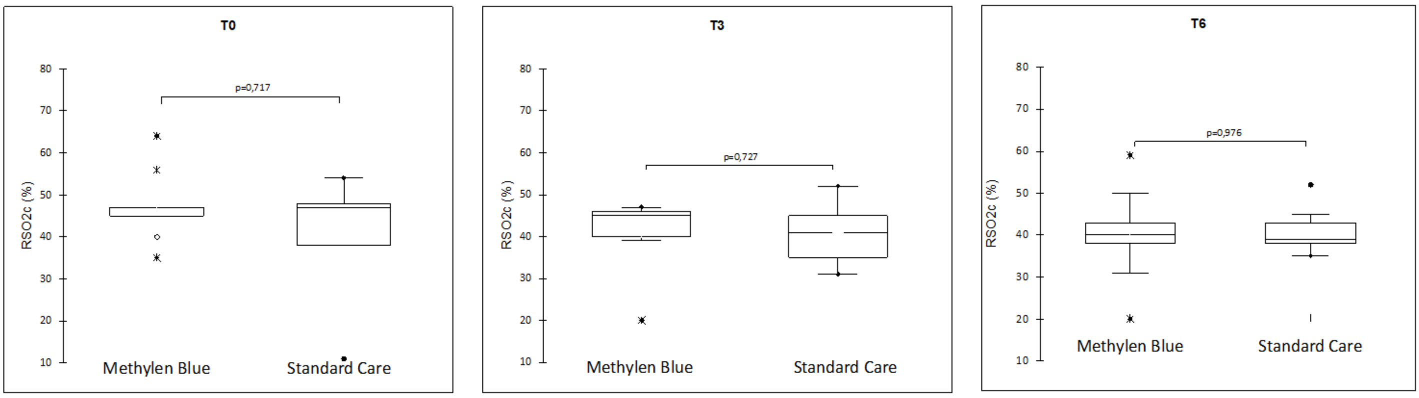

Figure S1. Regional cerebral tissue oxygen saturation (rSO2) in the right frontal lobe during the study. T_0: start of VA-ECMO; T_3: 3 hours after randomization; T_6: 6 hours after randomization. Data are presented as median (25th – 75th percentile).
